# Role of eccentricity in early Holocene African and Asian summer monsoons

**DOI:** 10.1038/s41598-021-03525-z

**Published:** 2021-12-16

**Authors:** Chi-Hua Wu, Shih-Yu Lee, Pei-Chia Tsai

**Affiliations:** Research Center for Environmental Changes, Academia Sinica, 128 Academia Road, Section 2, Nankang, Taipei, 115 Taiwan

**Keywords:** Atmospheric science, Palaeoceanography, Palaeoclimate

## Abstract

The effect of precession on paleoclimate changes depends on eccentricity. However, whether and to what degree eccentricity relates to millennial-scale monsoonal changes remain unclear. By investigating climate simulations with a fixed precession condition of 9 ka before the present, we explored the potential influence of eccentricity on early-Holocene changes in the Afro–Asian summer monsoons. Compared with the lower eccentricity of the present day, higher eccentricity in the early Holocene strengthened the continental summer monsoons, Pacific anticyclone, and Hadley circulation, particularly over the ocean. Over Africa, the eccentricity-induced “dry-gets-wetter” condition could be related to the Green Sahara, suggesting a superimposed effect of precession. Over the western Pacific, the tropical response to eccentricity may have been competitive in terms of what an extremely high obliquity may have caused. A downscaled modulation of eccentricity in relation to precession and obliquity cannot be ignored when paleomonsoon records are studied. Regarding early-Holocene monsoonal changes in South Asia, however, a high eccentricity may have had only a secondary effect on enhancing the monsoonal precipitation in the southern edge of the Tibetan Plateau, exhibiting the weak power of candle-like heating. This suggested that sizable monsoonal changes over the northern Indian Ocean and India–Pakistan region are unrelated to early-Holocene eccentricity.

## Introduction

Millennial-scale climate changes can be orbitally driven and are typically weighted between precession and obliquity^1^, with modulation in eccentricity^2,3^. As the perihelion shifts from boreal summer to winter solstice, seasonal insolation changes, resulting in variation of the Hadley circulation and subtropical anticyclones^4,5^, monsoons^6^, and tropical hydrological cycle^7,8^. In contrast to the direct effect of precession on tropical and subtropical regions, obliquity substantially influences meridional temperature gradients and glacial cycles^9–11^, and the effects can be seasonally and regionally dependent^12,13^. Studies have further suggested the potential cancellation of differential orbital effects; the precession minimum’s influence on the boreal summer monsoons may partially disappear when obliquity is extremely low^13,14^. Inevitably, regarding latitudinal energy distribution and seasonal dynamics, the changes in a paleoclimatology might have been influenced by distinct orbital forcings in combination.

Compared with the present day, summer insolation was much higher in the early Holocene as a result of the precession minimum, larger obliquity, and slightly higher eccentricity. Paleoclimatology proxies suggest that early-Holocene summer monsoons penetrated farther northward than their present-day counterparts do in West Africa, South Asia, and East Asia^15,16^. Climate modeling results have further indicated major changes in atmospheric circulation including the (1) strengthening and westward shift of all subtropical anticyclones, including the so-called South Asian High in the upper troposphere and western North Pacific High in the mid-to-lower troposphere; and (2) weakening and northward shift of the midlatitude westerly jet stream in the upper troposphere^17,18^. The orbitally driven circulation changes occurred broadly in latitudes in the early Holocene, implying the combined effects of precession and obliquity.

With a higher eccentricity than that in the early Holocene, the effect of precession and obliquity may have been pronounced in the Eemian. However, the role of eccentricity has not been well addressed in relation to Holocene and millennial-scale climate changes. Regarding the precessional index, identified by the combined effects of precession and eccentricity, whether the modulation of obliquity (and consequently, weighted orbital effects on monsoonal changes) by eccentricity was considerable remains unclear. Using time-slice simulations of orbital changes mostly obtained from previous studies, this study identified the potential effect of eccentricity on early-Holocene monsoonal changes. “Materials” describes the models, experimental simulations, and methods of identification. “Orbitally induced insolation changes” details the orbitally induced changes, and “Influence of eccentricity in the early Holocene” focuses on the orbitally induced changes in dry or wet patterns and subtropical atmospheric circulation during the Afro–Asian summer monsoon. “Summary and discussion” summarizes and discusses the findings.

## Materials

### Experimental simulations

We explored the modeling of monsoons in the early Holocene through time-slice experimental simulation at the fixed orbital condition of 9 ka before the present (BP; 9 K simulation), which had an approximate eccentricity of 0.019, obliquity of 24.2°, and perihelion precession of 312°. Time-slice simulations at the orbital condition of 1 ka after the present (AP; + 1 K simulation) and 31 ka BP (31 K simulation) were further analyzed; compared with present day, eccentricity of 1 ka AP is closer to that of 31 ka BP. With almost the same perihelion precession, the contrasting insolation between the 9 ka BP and 31 ka BP states resulted primarily from obliquity (24.2° vs. 22.3°) and eccentricity (approximately 0.019 vs. 0.016). We identified early-Holocene changes by comparing the 9 K and + 1 K simulations (H case); orbitally induced climate changes were related to the perihelion precession, larger obliquity, and higher eccentricity (also approximately 0.019 vs. 0.016). We further focused on the simulation with the same orbital condition as 31 K but with an obliquity of approximately 24.2° (31KO). A comparison between 31 and 31KO exhibited an extremely large contrast in obliquity (O case). An approximate effect of eccentricity through the Holocene could be evaluated through a comparison of 9 K and 31KO (EH case), in which the minimal difference in obliquity was neglected; with a difference of obliquity smaller than 0.02° (1% of obliquity change in the O case), a large difference in orbital-scale contrasts was not anticipated when using the sensitivity simulations. Despite that attribution bias could not be fully excluded, nonlinear response was omitted from consideration to demonstrate the signature of the O and EH cases. A total of four simulations investigated in this study were also used in other studies^19,20^ and are summarized in Table 1.

**Table 1:** Orbital parameters (*e* eccentricity; *p* precession; *o* obliquity) fixed in the H, O, and EH cases (unit of precession and obliquity is degree).

Case	Orbital forcing			Note
	e	p	o	
H (9 K minus + 1 K)	0.019 vs. 0.016	312 vs. 120	24.2 vs. 23.3	Through the Holocene
O (31KO minus 31 K)	0.016 vs. 0.016	312 vs. 312	24.2 vs. 22.3	Extremely large obliquity change
EH (9 K minus 31KO)	0.019 vs. 0.016	312 vs. 312	24.2 vs. 24.2	Eccentricity change through the Holocene

This series of orbitally related simulations were conducted using the Community Atmospheric Model (CAM) version 5.1 of the National Center for Atmospheric Research (NCAR) coupled with the slab ocean model (SOM) (http://www.cesm.ucar.edu). Since net heat transport by ocean current was prescribed in the CAM/SOM simulation, the effect of oceanic changes on monsoons cannot be considered. The simulations, with the boundary and initial conditions extracted from the Community Earth System Model (CESM) preindustrial control experiment^21^, were conducted at approximately 1° horizontal resolution and 30 vertical levels in the CAM^22^. The outputs for the integrated years 21–40 were analyzed.

### Identification of dry–wet condition and monsoon indices

In this study, a dry condition was identified when the averaged 5-day (pentad) precipitation was < 1 mm day^−1^, and a pentad precipitation ≥ 1 mm day^−1^ indicated a wet condition. Regarding the subseasonal evolution of monsoons, including the monsoonal phase transitions, the pentad has been widely considered a suitable unit^23^. We detected the frequency of dry or wet conditions between May and September (30 pentads in total for each year, i.e., wet + dry pentads). To quantify the orbitally induced changes in the summer monsoon circulation, we examined four dynamical indices. In terms of the combined effects of the southwesterly monsoon and tropical easterly jet stream on the West African monsoon, we assessed the strength of the monsoon based on the difference between the standardized values of the 925-hPa wind speeds minus the 200-hPa zonal winds over the region (20°W–20°E, 5°N–17.5°N)^24–26^. The strength of the Somali jet stream was assessed as the square root of the kinetic energy of the 850-hPa horizontal wind over the region (50°E–70°E, 5°S–20°N)^27^. We used the monsoon Hadley circulation to examine the South Asian summer monsoon, which is defined a meridional wind shear between 850 and 200 hPa over the region (70°E–110°E, 10°N–30°N)^28^. To quantify the monsoon trough strength over the western North Pacific (WNP), we calculated the difference in 850-hPa westerlies between regions (100°E–130°E, 5°N–15°N) and (110°E–140°E, 20°N–30°N)^29^.

### Insolation and proxy records

**Figure 1:**
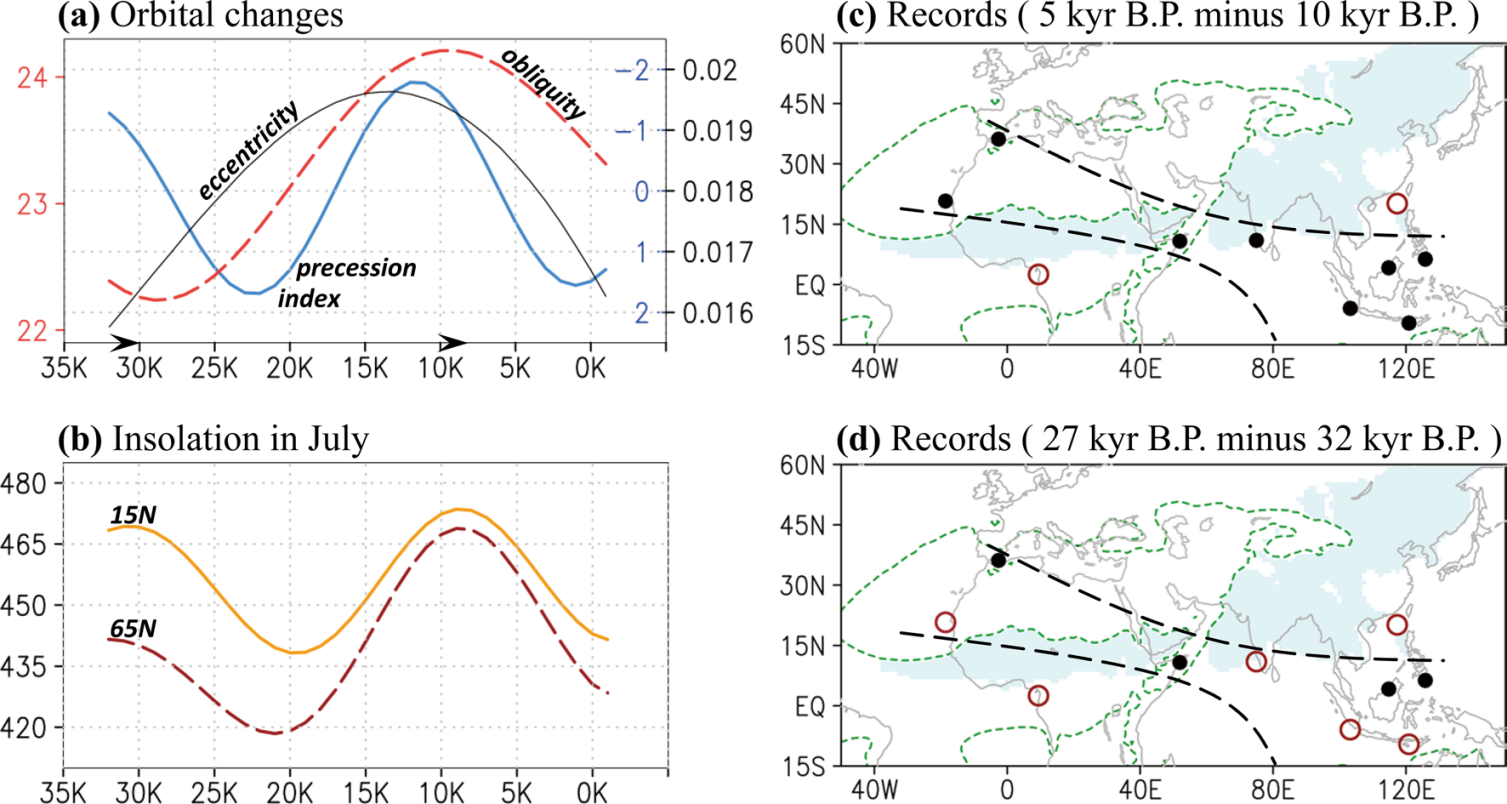
(**a**) Millennial-scale variation of precession index (blue), obliquity (red), and eccentricity (black) between 31 ka BP and 1 ka AP. (**b**) Solar insolation (W m^−2^) in July at 15°N (orange) and 65°N (red) between 31 ka BP and 1 ka AP. Isotope changes in the collected proxy records listed in Table in (**c**) 5 ka BP compared with 10 ka BP and (**d**) 27 ka BP compared with 32 ka BP (see identification in the “Materials” section). Red circles (black dots) correspond to the wet-to-dry (dry-to-wet) changes. Green contour lines denote that the climatological mean summer (May–September) precipitation is equal to 1 mm day^−1^ in 1 ka AP. Monsoon precipitation is also marked by shadings, identified when local summer precipitation is larger than 1 mm day^−1^ and exceeds 55% of annual total (similar to identification in Wang, et al.^47^). The software GrADS Version 2.1.1.b0 (http://cola.gmu.edu/grads/grads.php) was used to generate the figure.

The millennial-scale changes in orbital parameters and insolation in July at specific latitudes can be accessed at http://vo.imcce.fr/insola/earth/online/earth/earth.html^30^. We collected 10 proxy records including cave and marine core oxygen isotopes across the Afro–Asia region; the selected records were sufficiently long such that the dry–wet trend could be established through a comparison of the record data. Downloaded from the paleoclimatology data website of the National Oceanic and Atmospheric Administration (NOAA), the data were measured for precipitation and hydroclimate, obtained for the periods 33 ka BP to 26 ka BP and 11 ka BP to 4 ka BP (see further details of the relevant proxy data in Table 2). Before determining the difference between two paleoclimate states, we averaged the oxygen isotopes over 2000 consecutive years, centered at a given reference time, as illustrated in Fig. 1c,d; for example, the values at 10 ka BP were the average values from 11 ka BP to 9 ka BP.

**Table 2:** Location and resolution (averaged in the period) of the relevant proxy records.

Location^Reference^	LON(°E)	LAT(°N)	Period (ka BP, relative to 1950)	Temporal resolution (ka)
Northwest Africa^37^	− 18.58	20.75	117.89 ~ 0.52	0.37
Alboran Sea^38^	− 2.62	36.14	51.38 ~ 1.01	0.30
Gulf of Guinea^39^	9.40	2.50	155.42 ~ 0.36	0.17
Arabian Sea^40^	51.95	10.77	87.58 ~ 0.96	0.13
Northern Indian Ocean^41^	75.00	10.98	31.60 ~ 0.12	0.12
Eastern Tropical Indian Ocean^42^	103.25	− 5.94	130.88 ~ 0	0.74
Borneo^43^	114.83	4.15	161.50 ~ 0	0.12
East Asia^44^	117.38	20.12	41.13 ~ 0.01	0.06
Indo-Pacific Warm Pool^45^	120.92	− 9.59	35.24 ~ 0.47	0.11
Pacific Warm Pool^46^	125.83	6.30	67.59 ~ 0.10	0.08

Reference proxy records used in Fig. 1. The use of location in the reference paper was followed.

## Orbitally induced insolation changes

At 9 ka BP and 31 ka BP, the perihelion precession was nearly the same at approximately 312° (Table 1 and Fig. 1a). Consequently, when compared through time slicing, the insolation-induced contrast in climatology could be primarily attributed to differences in obliquity (24.2° vs. 22.3°) and eccentricity (0.019 vs. 0.016). Compared with that at 31 ka BP, the insolation in July at 65°N was much higher at 9 ka BP, and the insolation gradients became considerably smaller between the tropical and extratropical regions (e.g., 65°N compared with 15°N, Fig. 1b). We speculated a contrasting evolution of dry or wet patterns across the two paleoclimate states. As depicted in Fig. 1c, when the change from 10 ka BP to 5 ka BP was focused, speleothems indicated a “dry-gets-wetter” condition over North and East Africa and a “wet-gets-wetter” condition over southern India and the Maritime Continent. By contrast, a “wet-gets-dryer” condition occurred over West Africa and the South China Sea. Despite approximately identical evolution of precession from 10 ka BP to 5 ka BP, only a “wet-gets-dryer” pattern was consistently identified from 32 ka BP to 27 ka BP (Fig. 1d). Regarding the differential shifts in dry–wet patterns across the two states (i.e., 9 ka BP vs. 31 ka BP), in addition to a major control of obliquity, the question of whether eccentricity contributed to the insolation-induced climate change can be raised. The combined effects of precession and eccentricity were implied by the precessional index, but whether obliquity-induced climate change was also eccentricity-modulated remains unclear.

**Figure 2:**
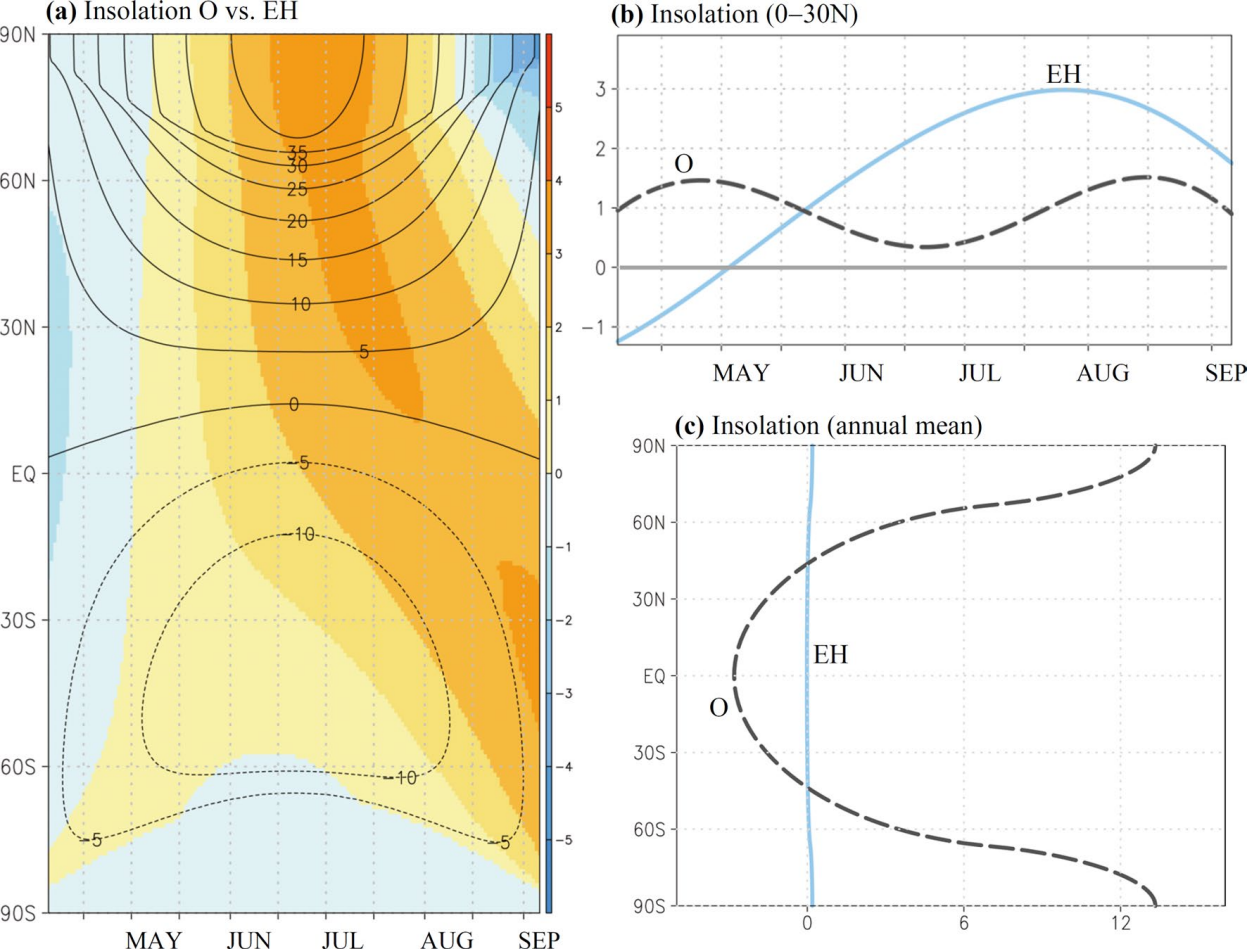
(**a**) Latitude–time distribution of global solar insolation (W m^−2^) for the O case (contours) and EH case (shaded). (**b**) Seasonal insolation evolution of Northern Hemispheric low latitudes (0°–30°N) for the O case (gray-dashed) and EH case (blue). (**c**) Annual mean insolation in latitudes for the O case (gray-dashed) and EH case (blue). The software GrADS Version 2.1.1.b0 (http://cola.gmu.edu/grads/grads.php) was used to generate the figure.

Climate modeling with seasonal response to orbital changes may illustrate dynamical and regional details regarding shifting dry–wet patterns. Before focusing on the atmospheric response to the orbital forcing, we examined seasonal insolation contrasts induced by obliquity and eccentricity individually. As presented in Fig. 2a, obliquity’s influence is seasonally systematic, centered on the summer solstice and primarily in extratropical latitudes. According to the modeling results from the O case, where the effects of extremely high and low obliquity were considered, the boreal summer insolation may be only slightly influenced in low latitudes. In regard to a fixed perihelion precession in the early Holocene, the insolation changes induced only by eccentricity (i.e., EH case) may depend on seasons, particularly in the tropical and subtropical regions, in which late summer is larger than spring and early summer (shadings, Fig. 2a). Notably, in boreal summer, the insolation changes induced by eccentricity and obliquity can be competitive in low latitudes (0°N–30°N), and July to August can exhibit better competition than in May (Fig. 2b).

Unlike the simulations with fixed obliquity in the O case (i.e. 24.2° vs. 22.3°), the minimal difference in obliquity was neglected in the EH case (with an approximate difference of 0.02° greater in 9 K than in 31 KO). We carefully calculated the annual average insolation contrast in the EH case (the precessional effect was removed). Compared with the annual insolation average in the O case, the effect of obliquity in latitudes was nonsignificant in the EH case (approximately zero, even in terms of the high latitudes, Fig. 2c).

## Influence of eccentricity in the early Holocene

### Dry–wet pattern shifts

**Figure 3:**
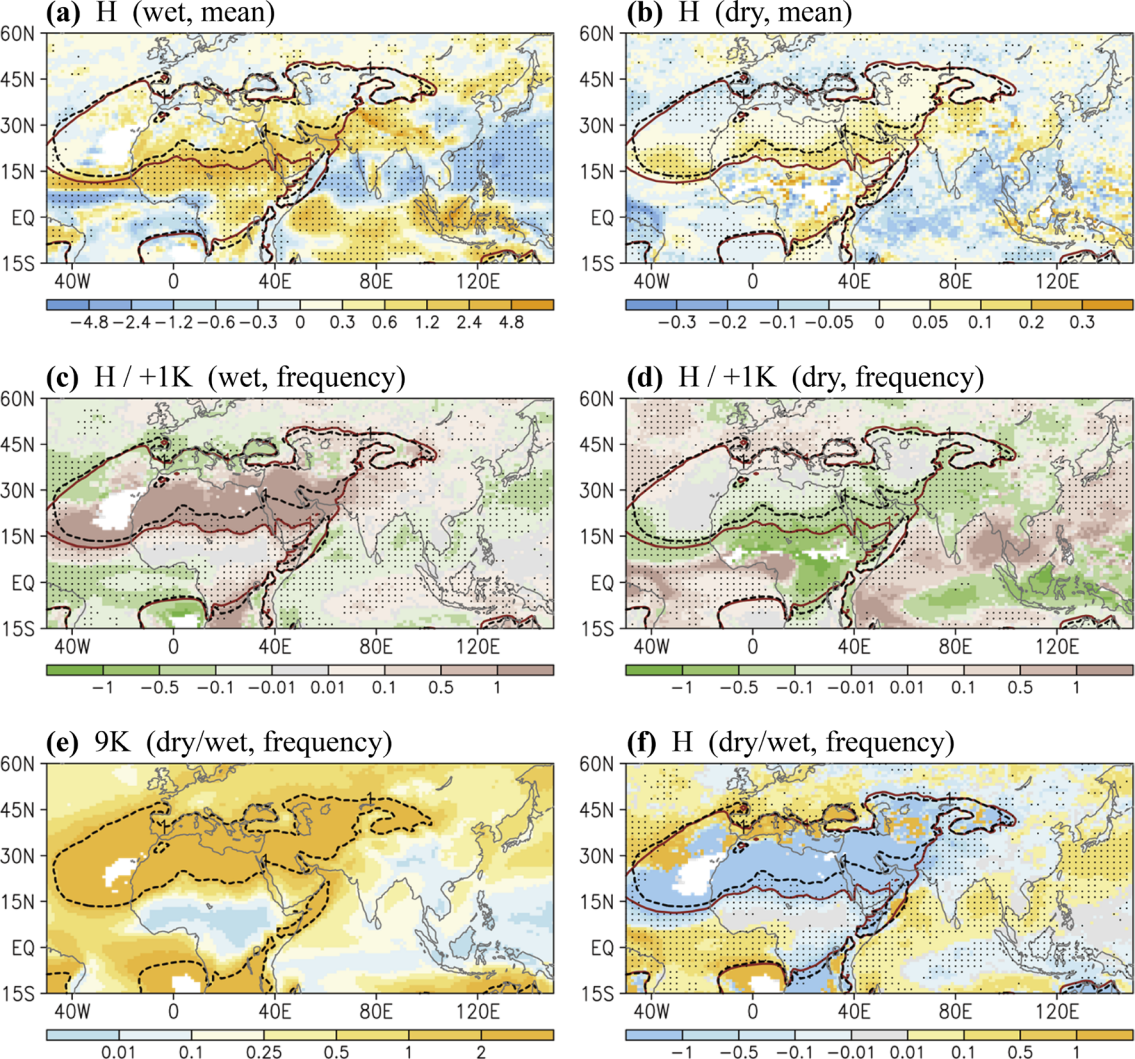
(**a**) Precipitation difference (shaded) of the wet condition in the H case. (**b**) Same as (**a**) except for the dry condition. (**c**) Difference in frequency (shaded, %) in the wet condition in the H case compared with that at + 1 K. (**d**) Same as (**c**) except for the dry condition. (**e**) Accounting rate of dry/wet in frequency in the 9 K simulation. (**f**) Rate of dry and wet condition frequency in the H case. Black and red contour lines denote that the climatological mean precipitation is equal to 1 mm day^−1^ in the 9 K and + 1 K simulations, respectively. Dots in (**a**–**d**,**f**) denote that the differences in mean precipitation and frequency have a confidence level of 90%. The software GrADS Version 2.1.1.b0 (http://cola.gmu.edu/grads/grads.php) was used to generate the figure.

Fundamentally, deserts and anticyclones can be closely related to the advancement of monsoons^31,32^. The border between monsoon and desert or anticyclone may be sensitive to systematic changes in large-scale circulation. Therefore, in addition to considering the wet condition that largely reflects monsoon precipitation, we examined the changes in continental dry regions. Compared with 1 ka AP, at 9 ka BP, the wet-gets-wetter condition was visible in most of the continental Afro–Asian monsoon region, equatorial Indian Ocean, and Maritime Continent (Fig. 3a); the wet-gets-dryer condition was observed over the subtropical oceanic regions, including the northern Arabian Sea, Bay of Bengal, South China Sea, and WNP. In regard to the pentads with a precipitation of less than 1 mm day^−1^ (i.e., dry condition), a reduction in precipitation was noted over the northern Arabian Sea, Mediterranean region, and midlatitude Atlantic Ocean (Fig. 3b); these areas with a “dry-gets-dryer” condition may correspond to the retreat of continental dry regions, including North Africa and the Arabian Peninsula.

Regarding the frequency of either the dry or wet condition, a large proportion (more than half) of changes occurred through the Holocene (H case) compared with the condition at 1 ka AP, particularly around the border of the continental dry area (Fig. 3c,d). The shifting dry–wet boundary was more clearly identified using the ratio of frequency between the dry and wet condition (i.e., dry/wet, Fig. 3e,f). The highlighted pattern that simultaneously detects changes in the dry and wet condition is useful for identifying the individual contribution of orbital forcing.

**Figure 4:**
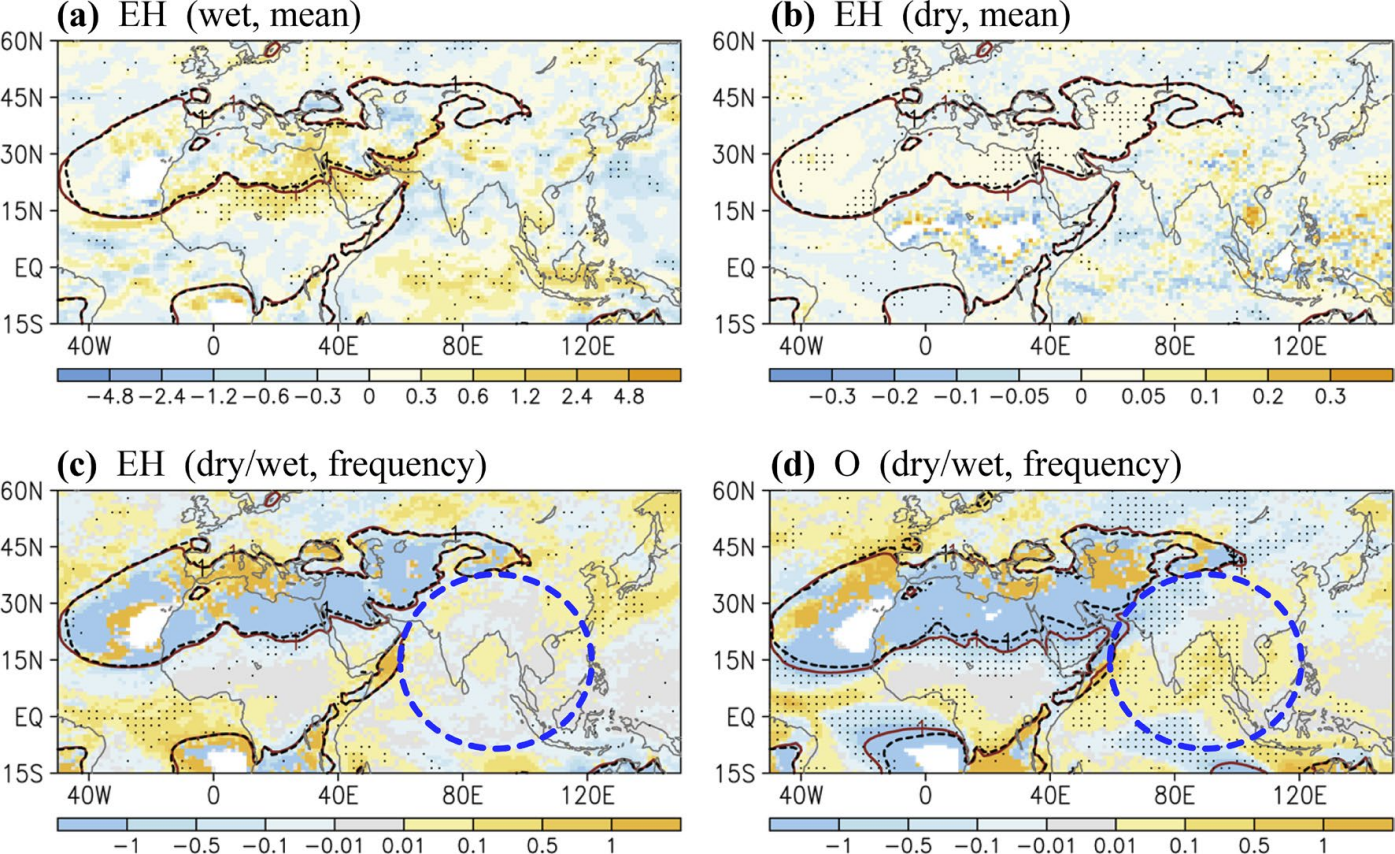
Same as Fig. 3 except for (**a**) Wet condition in the EH case; (**b**) Dry condition in the EH case; (**c**) Rate of dry and wet condition frequency in the EH case; (**d**) Rate of dry and wet condition frequency in the O case. In (**a**–**c**), black and red contour lines denote that the climatological mean precipitation is equal to 1 mm day^−1^ in the 9 K and 31KO simulations, respectively. In (**d**), black and red contour lines denote the mean precipitation equal to 1 mm day^−1^ in the 31KO and 31 K simulations, respectively. Dots denote that the differences in mean precipitation or frequency have a confidence level of 90%. Blue-dashed circles denote opposite changes over South Asia observed in the EH case compared with the O and H cases. The software GrADS Version 2.1.1.b0 (http://cola.gmu.edu/grads/grads.php) was used to generate the figure.

After the analysis of dry–wet pattern changes in the H case (Fig. 3), the potential influence of eccentricity was investigated, as depicted in Fig. 4. Although weaker in intensity and covering narrow regions, the dry–wet pattern shift in the H case was largely observed in the EH case, including the changing border of the continental dry area over North Africa and the Arabian Peninsula. Regardless of what an extremely large obliquity may have caused (O case, Fig. 4d), the dry-gets-wetter condition remained competitive in the EH case (Fig. 4c), suggesting eccentricity’s considerable contribution to early-Holocene climate change. In the EH case, the dry–wet condition changes were trivial in South Asia (Fig. 4c,d). Opposite changes were also observed in the EH case compared with the H case; for example, a higher eccentricity caused a reduction in precipitation over North India (Fig. 4a,b). Nevertheless, eccentricity-induced changes may have played a minor role in the early Holocene, though precession and obliquity may have cancelled the reversed effect of eccentricity (Figs. 3f and 4c,d). However, in regard to a paleoclimatology with large eccentricity or competing effects of precession and obliquity, the influence of eccentricity cannot be ignored.

### Changes in subtropical circulation and monsoons

Compared with 1 ka AP, at 9 ka BP, summer monsoons penetrated farther northward over the Afro–Asia continents, followed by a reduction in precipitation over subtropical regions (10°N–20°N) that corresponded to a stronger North Pacific High and, consequently, a suppressed monsoon trough over the WNP. The monsoon indices detailed in Table 3 (with August as the focus) quantify the approximate dynamical changes in the Afro–Asian summer monsoons. A comparison of the H and EH cases further suggested substantial eccentricity effects on the monsoon in West Africa (30%), Somali jet stream (27%), and WNP monsoon trough (24%), but changes in the monsoon Hadley cell over South Asia were likely negligible. In addition, consistent with changes in dry–wet patterns, the eccentricity-induced monsoon changes could be competitive in the contribution of obliquity (over West Africa, Somali jet region, and WNP condition; Table 3).

**Table 3:** Monsoon indices in August in the 9 K simulation, H case, EH case, O case, percentage of EH/H, and percentage of EH/O. The West Africa monsoon index has a normalized unit; unit of the other indices is m s^−1^.

	West Africa monsoon	Somali jet	Monsoon Hadley cell	WNP monsoon trough
9 K	2.38	10.81	4.98	1.18
H	1.12	− 1.66	1.80	− 6.41
EH	0.34	− 0.45	0.05	− 1.57
O	0.29	− 0.41	1.44	− 2.66
EH/H	30%	27%	3%	24%
EH/O	117%	110%	3%	59%

**Figure 5:**
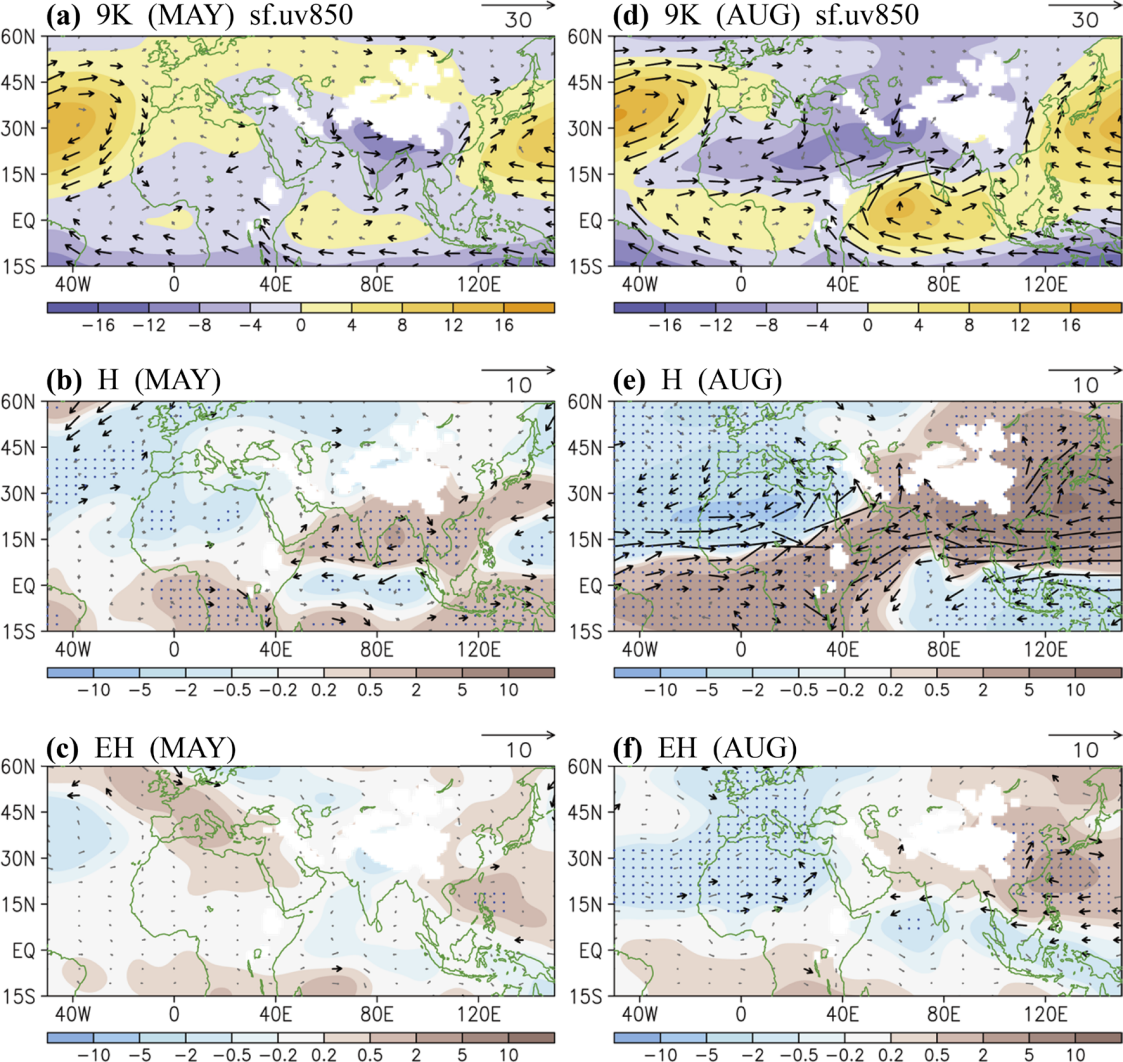
The 850-hPa streamfunction (shading, 10^6^ m^2^ s^−1^) and wind (vectors, m s^−1^) in May: (**a**) 9 K, (**b**) H, and (**c**) EH. (**d**–**f**) Same as (**a**–**c**) except for August. Wind vectors are indicated when the speeds are greater than 1 (climatology) and 1 (difference) m s^−1^. Dots in (**b**,**c**,**e**,**f**) denote that the differences in streamfunction have a confidence level of 90%. The software GrADS Version 2.1.1.b0 (http://cola.gmu.edu/grads/grads.php) was used to generate the figure.

**Figure 6:**
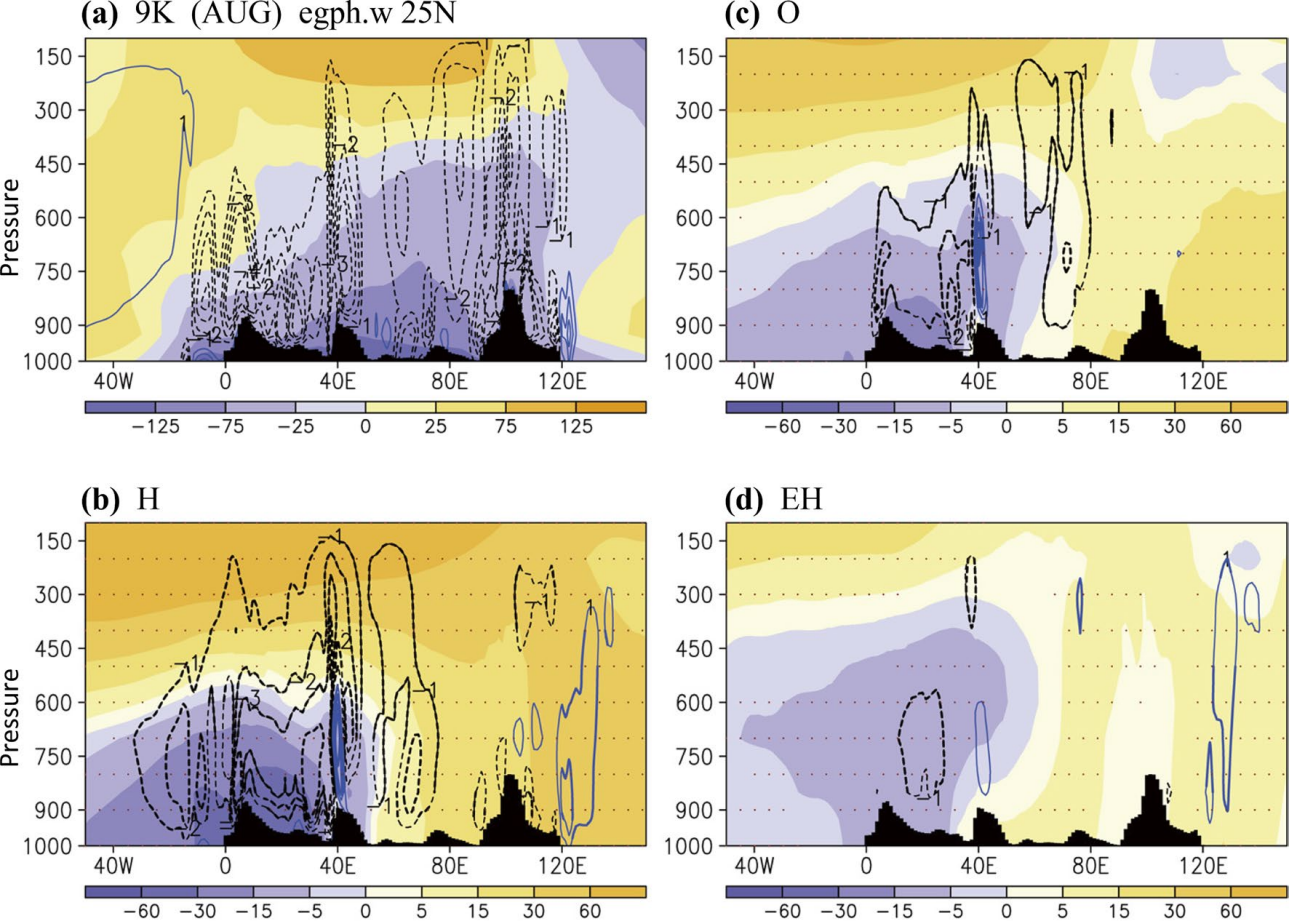
Geopotential heights (gpm) and omega (Pa min^−1^) in August along 25°N: (**a**) 9 K (zonal mean is subtracted), (**b**) H, (**c**) O, (**d**) EH. Black bars denote topography. Differences with a 90% confidence level are marked with dots (geopotential height) and thick contour lines (omega). The software GrADS Version 2.1.1.b0 (http://cola.gmu.edu/grads/grads.php) was used to generate the figure.

**Figure 7:**
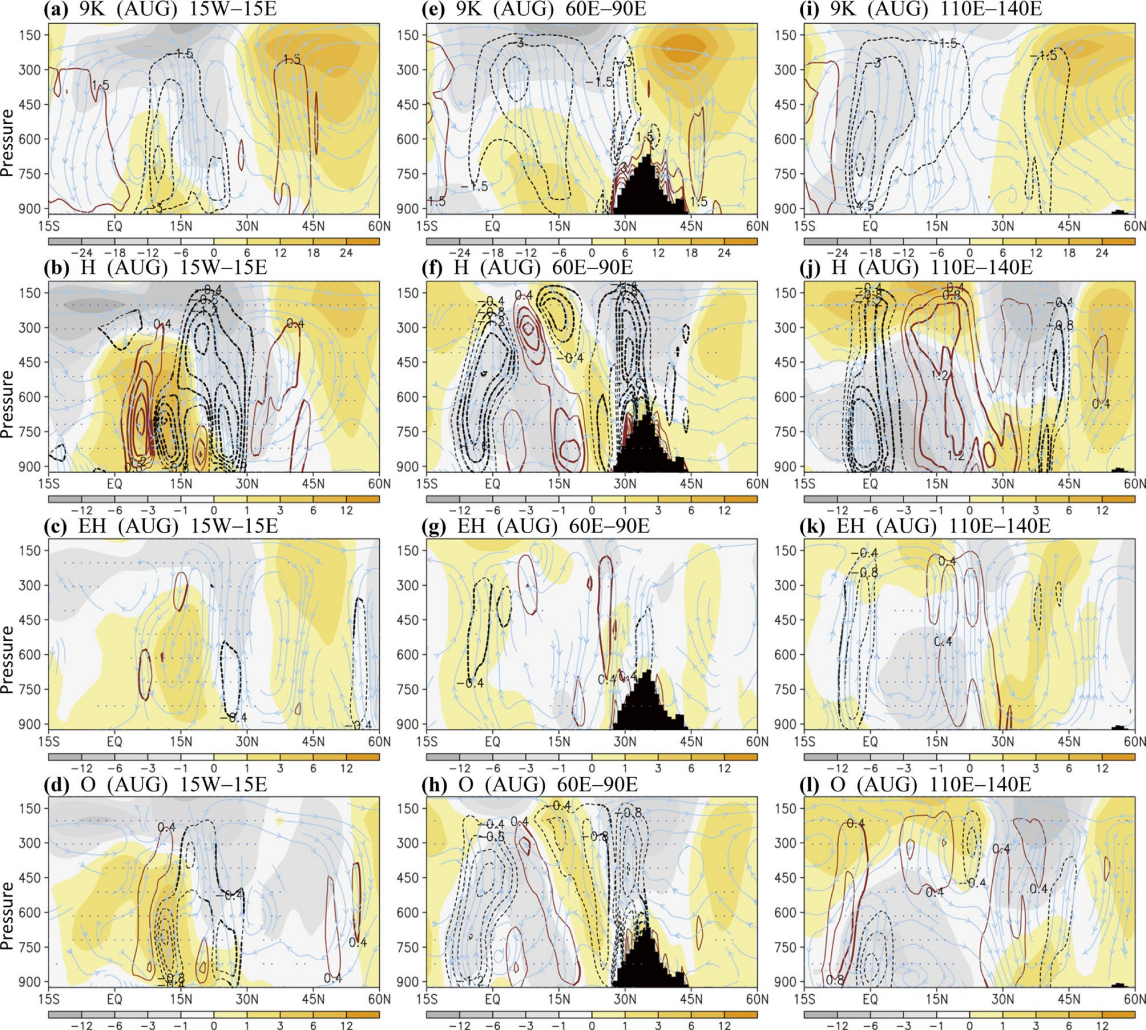
Meridional circulation in August in (**a**–**d**) 15°W–15°E, (**e**–**h**) 60°E–90°E, and (**i**–**l**) 110°E–140°E. The streamlines consist of divergent meridional winds (m s^−1^) and omega (Pa min^−1^, multiplied by − 1); color shadings denote the zonal winds (m s^−1^), and contour lines denote omega. Black bars denote topography. Upper to lower panels detail the 9 K, H, EH, and O conditions. Thick contour lines (dots) denote that the differences in omega (zonal winds) have a confidence level of 90%. The software GrADS Version 2.1.1.b0 (http://cola.gmu.edu/grads/grads.php) was used to generate the figure.

To provide an overview of large-scale circulation changes and explore the connection between monsoons, we investigated the orbitally induced changes in terms of lower-tropospheric streamfunction circulation, vertical distribution of the subtropical geopotential heights, and wind fields in detail in relation to the focused monsoon regions (Figs. 5, 6, 7). Corresponding with the seasonal characteristics of the Afro–Asian monsoon system, we respectively focused on the early-summer and late-summer monsoon periods. In May (presently in late May), summer monsoons begin in South Asia and East Asia (Fig. 5a); the approximately synchronous advancement of the monsoons closely relates to the evolution of the WNP High^33^. As the changes in the H case suggested, the monsoon low (negative streamfunction) weakened in subtropical South Asia, which corresponded to an expansion of the WNP anticyclone (positive streamfunction). The North Atlantic anticyclone in turn weakened, characterized by a decrease in positive streamfunction over the North Atlantic and North Africa (Fig. 5b). Consistent with the H case, although with a relatively weak signal (Fig. 5c), high eccentricity can induce the weakening of the North Atlantic anticyclone and strengthening of the North Pacific anticyclone.

In August, summer monsoons reached North Africa and West and South Asia, characterized by strong negative streamfunction (Fig. 5d) that corresponds to the northward evolution of the Somali jet stream. Compared with 1 ka AP, at 9 ka BP (Fig. 5e), the low-level streamfunction altered to form a dipole structure north at approximately 10°N (positive in Asia–Pacific vs. negative in Atlantic–Africa) and a reversed dipole pattern in the tropics south of 10°N. Corresponding to the streamfunction circulation changes, the monsoon southwesterly flows strengthened in West Africa, shifting the wet condition farther northward. In Asia, influenced by the weakening of the Somali jet stream and westward expansion of the WNP High, the westerly winds decreased at 10°N to 20°N and the southerly winds north of 20°N increased; the weakened westerly flow resulted in an increased dry condition (Fig. 3f), and the strengthened southerly flow favored the northward advancement of monsoon precipitation in South and East Asia (refer to Fig. 3a). Moreover, influenced by the weakening of the North Atlantic anticyclone, the northerly flow strengthened in the Mediterranean Sea and some regions of North Africa, which may be responsible for the dry-gets-dryer condition (Fig. 3f).

Regarding the influence of eccentricity only (EH case, Fig. 5f), the changes characterized by the dipole pattern in subtropical Africa and East Asia–WNP were considerable. Although some major monsoon-associated changes in the H case were not visible, including the tropical monsoon westerly in Africa and the Somali jet stream, a cyclonic (negative) streamfunction anomaly was induced by high eccentricity, which favors the wet-gets-wetter condition around the southern India and Sumatra region (also refer to Fig. 1c). We continued to examine the vertical distribution of the subtropical geopotential heights along 25°N in August, which exhibits more clearly observable changes than does May. Associated with the summer monsoon, low geopotential heights occurred from surface levels upward to the middle troposphere at approximately 450 hPa over South and East Asia (Fig. 6a), which corresponded to a deep intrusion of upward motion. The monsoon low geopotential heights could only reach the lower troposphere over West Asia and only surface levels over Africa, partly following the Sverdrup vorticity balance^32^. In the mid-to-lower troposphere, sizable anticyclones (high geopotential heights) over the Atlantic and Pacific were recorded. The monsoon and anticyclones could be further connected when coupled with an intercontinental-scale anticyclone in the upper troposphere centered over South Asia. The changes of these circulation systems have a large-scale and dynamical control of the dry–wet pattern shifts.

Associated with the strengthening of the monsoon in Africa and South Asia, the intercontinental anticyclone was stronger in the upper troposphere at 9 ka BP compared with 1 ka AP. Corresponding to an overall northwestward expansion of the Afro–Asian summer monsoon, the anticyclone in the mid-to-lower troposphere was stronger, expanding westward over the Pacific Ocean (H case, Fig. 6b). The geopotential height changes induced by a larger obliquity (O case, Fig. 6c) exhibited a shifting pattern similar to the changes in the H case, with a marked increase in upward air motion in Africa and South Asia. In the EH case with a high eccentricity (Fig. 6d), the decrease in geopotential heights was observed deeper in vertical from the lower troposphere up to approximately 400 hPa. Considerable changes in vertical motion were also recorded in Africa (increase in upward motion) and the WNP (increase in downward motion). The sizable enhancement of upward motion over South Asia (60°E–80°E) was not noted in the EH case, suggesting the differential effects of individual orbital forcing.

### Changes in meridional circulation across regional monsoons

In this subsection, by investigating detailed changes in wind fields (also focusing on August), we separately explored monsoon and meridional circulation in Africa (15°W–15°E), South Asia (60°E–90°E), and East Asia–WNP (110°E–140°E); the monsoon–midlatitude relationship was also examined. In Africa, corresponding to a northward penetration of monsoon in the H case, the low-latitude westerly flows strengthened in the lower troposphere, and the African Easterly Jet (AEJ) shifted northward at approximately 700 hPa, with the upward motion greatly enhanced in the region north of the AEJ (Fig. 7a,b). An enhancement of the Northern Hemisphere Hadley cell was further identified, which was dynamically consistent with the strengthening of the upper-tropospheric anticyclone (as illustrated by the geopotential heights in Fig. 6b and zonal wind in Fig. 7b). In regard to the influence of eccentricity (Fig. 7c) or obliquity (Fig. 7d), the major changes in the H case may have been induced by both, including the enhancement of tropical westerly flow. However, differential influence caused by obliquity, as opposed to eccentricity, was observed. Characterized by the anticyclonic circulation response to the enhanced convective activity over the subtropical region (Fig. 7c,d), obliquity influences high latitudes through a major Hadley-style circulation response, whereas eccentricity may influence mid-to-high latitudes by triggering wavy fluctuation.

Compared with 1 ka AP (H case), in South Asia at 9 ka BP, convection was suppressed at 10°N to 20°N as a result of the enhanced convection over the tropics and southern slope of the Tibetan Plateau–Himalayas (Fig. 7e,f); the topographically driven heating markedly influenced the monsoon circulation and can be described as a candle-like heating^34^. In the early Holocene, unlike the essential obliquity contribution (Fig. 7h), the enhancement of candle-like heating was irrelevant to eccentricity (Fig. 7g). In East Asia–WNP, corresponding to the meridional expansion of the WNP High, convection was greatly enhanced around the equator and midlatitude East Asia (Fig. 7i,j). Over this coastal and oceanic area, the meridional circulation change induced by eccentricity was considerable in the early Holocene (Fig. 7k), with a major contribution to the lower latitudes (10°S–30°N). A comparison of the H, EH, and O cases (Fig. 7j–l) further indicated that eccentricity and obliquity may have considerable effects on the oceanic monsoon in the early Holocene (e.g., regarding the effect on lower latitudes vs. the midlatitude region; 30°N–45°N).

In terms of the changes in the midlatitude westerly jet stream over all focused regions (Fig. 7), a northward shift with an enlarged northeast-southwest tilt of the jet stream was observed in the H case, with the jet stream shifting considerably northward in the O case; almost no shift of the jet stream was noted in the EH case. These contrasting shifts of the midlatitude westerly jet stream follow the differential meridional insolation gradients (markedly larger in the O case compared with those in the EH case, Fig. 2a). The contrasting shifts of the jet stream again suggested the competing effects of a large obliquity tilt and high eccentricity. In the early Holocene, high eccentricity can strengthen the midlatitude westerly jet stream, and high obliquity can shift the jet stream farther northward.

## Summary and discussion

**Figure 8:**
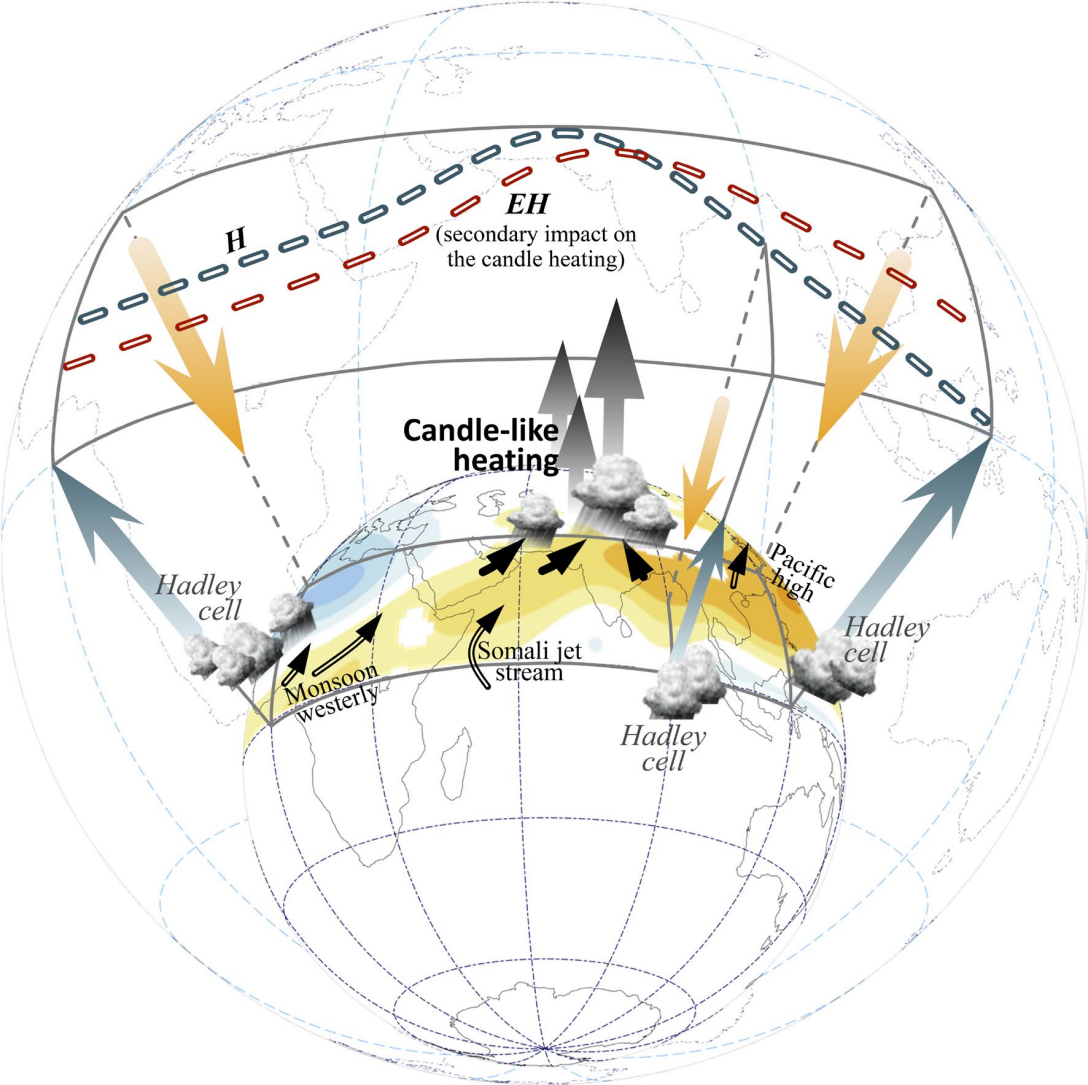
Schematic of the Afro–Asian summer monsoon changes in the early Holocene (H case) and potential eccentricity contribution only (EH case). Compared with the present, the Hadley circulation strengthened, WNP lower tropospheric anticyclone expanded, and continental Afro–Asian monsoons shifted farther northward in the early Holocene (shadings denote the 850-hPa streamfunction change in the H case). Corresponding to the northward advancement of the monsoon in South Asia, the enhanced monsoon precipitation over the southern slope of the Himalayas resembles candle-like heating and, combined with the enhanced Hadley circulation, suppressed monsoon precipitation in 10°N–20°N. Influenced by a high eccentricity (i.e., EH case), the enhancement of the Hadley circulation and strengthening of the African monsoon and WNP anticyclone can be clearly identified, although with a relatively weak signal. By contrast, eccentricity exhibits no considerable effect on the candle-like heating over South Asia. Consequently, a northward shift of the South Asian monsoon is not visible. The software GrADS Version 2.1.1.b0 (http://cola.gmu.edu/grads/grads.php) and G.Projector Version 3.0.2 (https://www.giss.nasa.gov/tools/gprojector/) were used to generate the figure.

In this study, we explored the role of eccentricity in modulating changes in the Afro–Asian summer monsoons in the early Holocene. We investigated the climate sensitivity of the modeled monsoons to the approximate eccentricity forcing during the Holocene (EH case). We further identified the potential contribution of eccentricity in this period (compared with the EH and H cases). As summarized in Fig. 8, because of the extremely high summer insolation in the early Holocene, the Hadley circulation was enhanced and the continental Afro–Asian monsoons shifted to latitudes farther north than their present-day condition. The increase in thermal heating over the Tibetan Plateau and monsoon precipitation over the southern slope of the Tibetan Plateau–Himalayas (also known as candle-like heating^35^) had a dominant effect on strengthening the South Asian monsoon circulation. With the combined effects of the enhanced tropical convection and northward shift of the continental monsoons, a wet-gets-dryer condition in the subtropical regions (10°N–20°N) was triggered. The suppressed monsoon precipitation over the Indochina Peninsula and South China Sea also corresponded to the expansion of the WNP High.

When the effect of high eccentricity only was analyzed, the overall enhancement of the Hadley circulation was identified, although with a relatively weak signal. The high eccentricity-induced strengthening of the African monsoon and WNP anticyclone remained considerable. Notably, the sizable enhancement of candle-like heating in the H case may be irrelevant to eccentricity. In the EH case, a wet-gets-dryer condition may occur over the North India–Pakistan region, rather than a wet-gets-wetter condition as noted in the H case. This implies a secondary effect of the Somali jet stream on the continental monsoon over South Asia.

In the East Asia–WNP sector, we noted an obvious contrast in the changes induced by obliquity versus those induced by eccentricity in the early Holocene. Obliquity influenced high latitudes through a major Hadley-style circulation response, whereas eccentricity may have influenced mid-to-high latitudes by triggering wavy fluctuation. Regardless of the influence of precession, we hypothesized that higher eccentricity strengthened the midlatitude westerly jet stream in the early Holocene, whereas the higher obliquity shifted the midlatitude jet stream farther northward.

Although eccentricity could play a minor role in the early Holocene, we proposed a downscaled effect of eccentricity on precession and obliquity. In a paleoclimatology with a large eccentricity, competing effect of precession and obliquity, and dominance of obliquity-paced glacial-interglacial cycles^36^, the role of eccentricity in modulating monsoon changes cannot be ignored. The modulation of eccentricity could be incorporated into an experimental simulation to study the climate effect of precession and obliquity individually. However, without considering the sensitivity simulations including model dependence, bias in our attribution analysis cannot be ruled out. Further investigation of data-model analysis, with different precession and obliquity conditions, should ensue to obtain a complete understanding of the downscaled modulation of eccentricity.
